# Advances and Prospects for Hydrogel-Forming Microneedles in Transdermal Drug Delivery

**DOI:** 10.3390/biomedicines11082119

**Published:** 2023-07-27

**Authors:** Xiaolin Hou, Jiaqi Li, Yongyu Hong, Hang Ruan, Meng Long, Nianping Feng, Yongtai Zhang

**Affiliations:** 1 Department of Pharmaceutics, Shanghai University of Traditional Chinese Medicine, No. 1200 Cailun Road, Pudong New Area, Shanghai 201203, China; houxiao_lin2008@163.com (X.H.); alijiaqi0@163.com (J.L.); 18221817885@163.com (H.R.); longmeng0608@126.com (M.L.); 2 Xiamen Hospital of Chinese Medicine, No. 1739 Xiangyue Road, Huli District, Xiamen 361015, China; 13906055433@126.com

**Keywords:** hydrogel-forming microneedles, transdermal drug delivery, controlled release, permeation pathway, environmental response

## Abstract

Transdermal drug delivery (TDD) is one of the key approaches for treating diseases, avoiding first-pass effects, reducing systemic adverse drug reactions and improving patient compliance. Microneedling, iontophoresis, electroporation, laser ablation and ultrasound facilitation are often used to improve the efficiency of TDD. Among them, microneedling is a relatively simple and efficient means of drug delivery. Microneedles usually consist of micron-sized needles (50–900 μm in length) in arrays that can successfully penetrate the stratum corneum and deliver drugs in a minimally invasive manner below the stratum corneum without touching the blood vessels and nerves in the dermis, improving patient compliance. Hydrogel-forming microneedles (HFMs) are safe and non-toxic, with no residual matrix material, high drug loading capacity, and controlled drug release, and they are suitable for long-term, multiple drug delivery. This work reviewed the characteristics of the skin structure and TDD, introduced TDD strategies based on HFMs, and summarized the characteristics of HFM TDD systems and the evaluation methods of HFMs as well as the application of HFM drug delivery systems in disease treatment. The HFM drug delivery system has a wide scope for development, but the translation to clinical application still has more challenges.

## 1. Introduction

Transdermal drug delivery (TDD) is a route of drug delivery for treating or preventing disease by absorbing drugs through the skin, permeating into the skin and further into the blood circulation [1]. TDD avoids first-pass effects, prolongs the action of drugs with short half-lives through slow release and avoids fluctuations in blood levels, reduces side effects and improves patient compliance [2]. The stratum corneum barrier plays a key role in TDD, and many methods have been used to improve the efficiency of TDD, including the use of chemical penetration enhancers and different physical enhancement approaches, such as microneedling [2,3], iontophoresis [4], electroporation [5], laser ablation [6] and ultrasound facilitation [7,8,9].

In recent years, microneedles have gained widespread interest in TDD and have shown brilliant achievements in delivering both chemical small molecules and biomacromolecules whilst being minimally invasive and painless [10,11,12,13,14,15,16,17]. Microneedles usually consist of micrometer-sized needles (50–900 μm in length) in the form of microneedle arrays that can successfully penetrate the stratum corneum and deliver drugs in a minimally invasive manner below the stratum corneum without damaging blood vessels and nerves in the dermis [18,19], improving patient compliance and allowing drugs exposed in the epidermis or dermis to be rapidly absorbed by surrounding capillaries and lymph nodes [20,21,22]. Some of the microneedle products approved for marketing by the FDA and undergoing clinical trials are shown in Table 1 and Table 2, respectively. As shown in Table 1, microneedling devices are often intended for aesthetic use rather than medical purposes. Additionally, clinical trials (Table 2) of microneedles for disease treatment, such as influenza, psoriasis, and diabetes, have gradually increased in recent years.

**Figure 1:**
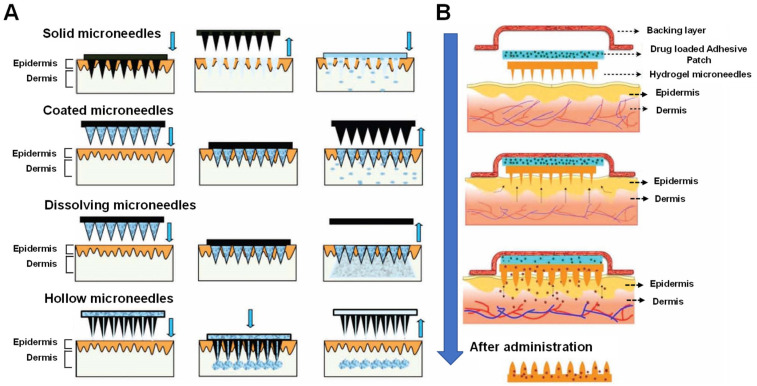
Schematic representation of methods of traditional (**A**) and hydrogel (**B**) microneedles mediated drug delivery across skin (arrows point to the order of operations). The figure was adopted from ref. [11] with permission from WILEY-VCH VERLAG GMBH & CO. KGAA.

Microneedles can be fabricated with different materials and can be classified into five main types (Figure 1), namely solid microneedles, coated microneedles, hollow microneedles, dissolving microneedles and hydrogel-forming microneedles (HFMs) [23,24,25]. Among these, HFMs, an attractive type of microneedles first reported in 2012, consist of a swellable polymer (cross-linked hydrogel) that enables the sustained delivery of drugs for long periods of time by either incorporating the drug into the polymer structure during preparation or by loading the drug into a separate reservoir and attaching it to the HFMs [26]. In the following, the application and evaluation methods of HFMs in TDD are analyzed and discussed in detail.

## 2. Characteristics of HFMs as TDD System

HFMs are safe, have no residual matrix material and are suitable for long-term, multiple-drug delivery. HFMs have the advantages of resisting the closure of skin tissue pores after puncture into the skin, and no matrix material remains when the HFM patch is removed due to the inherent swelling insolubility and viscoelastic properties of the matrix material [26,27]. HFMs also bring benefits from avoiding drug deposition after microneedle tip penetration [28].

HFMs are characterized by water absorption and swelling, and sustainable and controlled drug release. Drugs can be loaded in HFMs in two ways, either by incorporating the drug into the microneedle matrix during preparation or by loading the drug into a separate reservoir and then attaching it to the hydrogel microneedle as a substrate [29]. Both methods of preparation allow for the continuous delivery of the drug over a long period of time. Materials used to prepare HFMs are non-toxic, degradable and biocompatible [30], and commonly used materials include natural compounds such as gelatin and polymer copolymers such as poly (methyl vinyl ether-co-maleic acid) cross-linked with polyethylene glycol (PMVE/MAPEG) [10,31]. Among these, PMVE/MAPEG has an excellent water absorption capacity and allows the preparation of super-swollen HFMs that can absorb fluids and swell up to 20 times their original size [11,32]. HFMs pierce the skin and rapidly absorb interstitial fluid, causing the hydrogel to swell, creating a continuous, unobstructed hydrogel conduit for the drug permeating into the skin [33].

HFMs are able to control the drug release behavior through the crosslinking density of the hydrogel microneedle matrix material, thus achieving controlled drug delivery kinetics [34]. For example, the degree of swelling of PMVE/MAPEG decreases with increasing cross-linkage. When the PMVE/MAPEG ratio was 2:1 and 4:3, respectively, the degree of swelling increased by 294% and 250%, respectively [35]. In addition, increasing the concentration of the cross-linking agent ethylene glycol dimethacrylate (EGDMA) decreases the release of the loaded drug, and the t_1/2_ of the drug increased from 2.64 h to 45.67 h when EGDMA was added from 1% to 8%. It indicates that by increasing the cross-linking agent, the cross-linking degree can be increased and the swelling degree can be decreased, which results in lower and more sustained drug release [36]. Some main characteristics of different HFM formulations have been summarized in Table 3.

## 3. Materials for Forming HFMs

Similar to other types of microneedles, the common preparation method for HFMs is mainly micromolding [43,44], where a microneedle matrix in its flowing hydrogel state is injected into the mold using centrifugal and decompression methods and then dried. The release and permeation behavior of the active ingredients is mainly controlled by the nature of the polymers that make up the microneedle matrix, independent of the microneedle preparation process.

Preferred microneedle materials should be biocompatible and non-immunogenic, have fine-mechanical strength, and be able to carry potentially large and complex drugs without damage. The commonly used materials for HFMs include Gantrez S-97, a co-polymer of poly(methylvinylether co. maleic acid) (PMVE/MA) [12,45], methacrylated hyaluronic acid (MeHA) [46], Gantrez AN-139, a co-polymer of poly(methylvinylether co. maleic anhydride) (PMVE/MAH) [47,48], polyvinyl alcohol (PVA)/polymer blends [49,50] and crosslinked PVA [51], crosslinked 2-hydroxyethyl methacrylate (pHEMA) [39], poly(styrene-b-acrylic acid) (PS-*b*-PAA) [52], modified silk [2] and clay/polymer blends [53].

**Figure 2:**
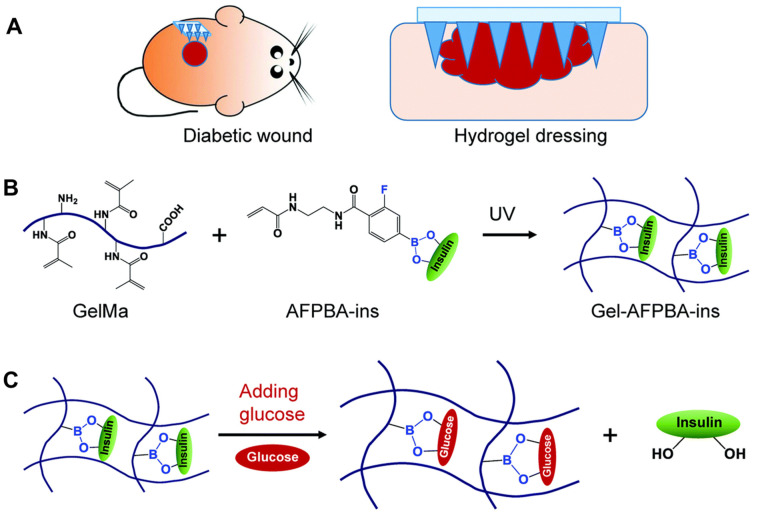
Schematic representation of responsive microneedle dressing for diabetic wound healing. (**A**) Diabetic wounds in mice treated with the hydrogel-based microneedle dressing. (**B**) Preparation of glucose-responsive insulin-releasing Gel–AFPBA–ins hydrogels. (**C**) Mechanism of glucose-responsive insulin release from the prepared hydrogels. The figure was cited from ref. [54] with permission from the Royal Society of Chemistry.

**Figure 3:**
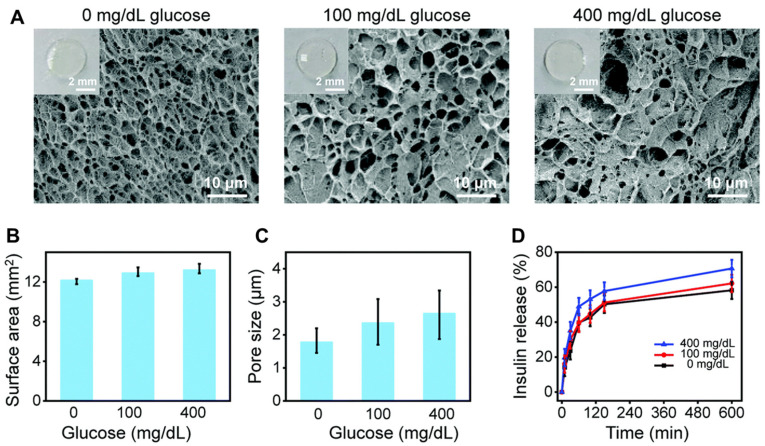
Schematic representation of glucose responsiveness and insulin release of the Gel–AFPBA–ins hydrogels. (**A**) Scanning electron microscope images of the cross-sectional morphology of the hydrogels after reaction with different glucose solutions. The insets show the appearance of the corresponding hydrogels. (**B**) Surface area and (**C**) average pore size of the hydrogels after reaction with different glucose solutions. (**D**) Insulin release kinetics from the hydrogels in different glucose solutions. Mean ± s.d. (*n* = 5 for 3 repetitions). The figure was cited from ref. [54] with permission from the Royal Society of Chemistry.

Some cross-linked polymers, such as those prepared through esterification reactions, have different degrees of polymerization, resulting in widely varying structures and therefore different properties of water absorption and swelling. This results in different rates of swelling after the microneedle tips are inserted into the skin, leading to different drug release profiles, such as burst and sustained drug release [11]. For example, HFMs based on the ‘super-swelling’ polymer PMVE/MAPEG consisted of drug reservoirs and microneedle tips which did not contain the drug [11]. When the tips were inserted into the skin, the microneedle tips rapidly absorbed the interstitial fluid, creating continuous conduits between the dermal microcirculation and the attached patch-type drug reservoirs, which sustainedly released the drug. In another report, HFMs (two layers) assembled from a lyophilized drug reservoir layer and a microneedle layer consisting of 20% (*w*/*w*) poly(methyl vinyl-maleic acid) crosslinked by esterification with 7.5% (*w*/*w*) poly(ethylene glycol) (Mw 10,000 Da) significantly enhanced the penetration of metformin hydrochloride in subcutaneous neonatal pig skin in vitro. The combined HFMs delivered 27.6-fold and 71.2-fold more drug over 6 h and 24 h, respectively, than controls using only drug reservoirs [12]. In addition, on-demand drug release can be achieved using HFMs through stimulation control. Hardy et al. prepared HFMs using the light-responsive materials 2-hydroxyethyl methacrylate (HEMA) and ethylene glycol dimethacrylate (EGDMA) to achieve on-demand delivery of ibuprofen [39]. In another report, the glucose-sensitive molecule 4-(2-acrylamidoethylcarbamoyl)-3-fluorophenylboronic acid (AFPBA) was chosen for integration with the hydrogel scaffold for its suitable equilibrium association constant with glucose (Figure 2C). After immersion in a glucose solution, the pore size of Gel-AFPBA-ins hydrogel (a hydrogel in which GelMA and AFPBA are cross-linked and then copolymerized with insulin) (Figure 2B) increased and insulin was released faster and more with an increasing glucose solution concentration (Figure 3) [54]. 

## 4. Evaluation Methods for HFMs

The materials, design, and preparation process of HFMs are important parameters in determining the properties of microneedles, while effective drug delivery also depends on the mechanical strength, skin penetration and release kinetics of HFMs.

### 4.1. Appearance and Morphology

The morphology and dimensions of HFMs (including the tip radius, height, width, length and spacing) can be characterized by optical microscopy, scanning electron microscopy (SEM) or optical coherence tomography (OCT), confocal laser scanning microscopy (CLSM), and multiphoton microscopy (MPM).

Optical microscopy and SEM are commonly used to image and measure the morphology of HFM arrays and the height, width and spacing of microneedles [55]. OCT imaging is highly accurate, has a certain imaging depth and imaging speed, and is often used to observe, in situ, the penetration depth of microneedle patches after puncturing into isolated or in vivo skin or to record the process of microneedle changes within the skin [56,57]. By loading the microneedles with fluorescent dyes similar to the physicochemical properties of the drug, the distribution of the drug in the microneedles can be assessed by CLSM [58].

### 4.2. Swellability and Water Insolubility

The swelling properties of HFMs were determined by placing the microneedle array in distilled water or PBS and removing and weighing at specific time intervals to calculate the percentage of swelling [59]. Ex vivo skin such as porcine skin was also used, with the subcutaneous tissue layer carefully removed and the skin placed on tissue paper equilibrated with PBS (pH 7.4). HFM patches were punctured into the isolated skin and then removed at specific time intervals and their base-width swelling capacity was measured using digital microscopy [60].

Water insolubility is an important property of HFMs. The solubility of HFMs was calculated by swelling them sufficiently and then placing them at 90 °C to dry completely to a constant weight and comparing the weight before swelling with the weight after swelling and drying to a constant weight [59].

### 4.3. Mechanical Strength

The shape of the microneedle determines how much force can be applied to the microneedle before the needle breaks. The diameter and angle of the needle tip, as well as the height and basal measurement of the microneedle, determine whether the microneedle can be safely and reliably inserted into the skin [61]. In general, smaller tip diameters, smaller tip angles and higher tip height-to-width ratios facilitate successful skin penetration. It was found that the average depth of penetration, as determined for the nine microneedles, was significantly higher for the triangular and square base geometries, (340 μm and 343 μm, respectively) than for microneedle arrays with a hexagonal base (197 μm). Accordingly, the average distance between the microneedle base plate and the stratum corneum was estimated at 660 μm, 657 μm and 803 μm for the triangular (34% penetration), square (34% penetration) and hexagonal (20% penetration) base geometries, respectively [62]. Mechanical strength is generally tested using a texturizer or a motorized force-measuring table [63,64]. For fracture testing, arrays of microneedles are microscopically observed before and after testing to determine height differences.

### 4.4. Skin Piercing and Transdermal Permeation Properties

Microneedles act on the skin surface, puncturing the epidermis and creating microscopic pores through which the drug diffuses into the dermal microcirculation. The success of microneedle puncture can be assessed using a paraffin membrane or porcine skin. The porcine skin has similar physical properties to human skin and can be used as a simulated human skin model [63,65,66]. When conducting relevant experiments, the skin was first washed with PBS (pH 7.4), and then the skin was placed dermally downwards on a wax sheet [67]. The HFMs were then pressed into the skin with the thumb for 30 s. The microneedle arrays were removed from the skin and stained with 150 µL of 1% methylene blue solution for 5 min to assess the position of the stained microneedle pinholes. Excess staining solution was gently washed away with PBS. The stained skin was imaged with a digital microscope and the percentage of stained blue microneedles was calculated to assess the skin puncture performance of the microneedle arrays. The 100% success rate indicated that all microneedle arrays would be observed in the skin [68]. In general, parameters such as the microneedle tip diameter, basal width, length, type of microneedle and its mechanical strength play a crucial role in forming the size of the microchannel in the skin [69].

OCT can be used for in situ observation of the depth of microneedle puncture into the skin in vitro. Kaiyue et al. inserted microneedle patches into rat skin in vitro and imaged the microneedle patches together with the treated skin with OCT; the microneedle tips reach a depth of about 300 μm into the skin and do not break during the insertion process [58].

Fluorescence microscopy can be used to examine the distribution and accumulation of the drug in the skin. Using fluorescence imaging, Aljuffali et al. observed that after transdermal administration, fluorescence was only detected on the skin surface in the free fluorescent probe group and only a weak fluorescent signal was present in the hair follicles, whereas fluorescence was significantly enhanced in the skin of the fluorescently labelled nanocarrier group, suggesting a pro-permeation effect of the nanocarriers [70].

When the drug itself is fluorescent or the drug delivery system is labelled with fluorescence, CLSM is often used to observe fluorescence at different skin depths, allowing verification of the depth of penetration of the agent into the skin tissue and visualization of the accumulation of the agent in the skin tissue. Alvarez-Roman et al. used CLSM to determine the penetration, distribution and accumulation of polymeric nanoparticles in isolated porcine skin [71]. Moreover, by loading coumarin 6 and rhodamine B into the inter-layer and tip-layer of the microneedles, respectively, and inserting into the skin of the knee joint of hairless rats for 30 min, the depth and distribution of the microneedles in the skin were evaluated by tracing the fluorescence of coumarin 6 and rhodamine B by using CLSM and performing 3D reconstruction [58].

MPM is suitable for the characterization of human skin and allows the assessment of skin morphology and layers at a subcellular level. The two-photon excitation principle overcomes the limitations of fluorescence imaging and allows for in vivo non-toxic manipulation. Excitation occurs almost exclusively at the target inspection site without damaging surrounding tissue [72]. MPM also extends the applicability of fluorescence lifetime imaging microscopy (FLIM), and MPM-FLIM allows non-invasive, high-resolution examination of human skin for in vitro, ex vivo, and even clinical in vivo applications [73]. MPM has been used to evaluate the pathophysiological features of inflamed skin, skin permeation and delivery of drugs [74,75,76].

### 4.5. In Vitro Release and Transdermal Behaviour

The in vitro TDD can be assessed by Franz diffusion in the donor compartment of the diffusion cell, with the stratum corneum of porcine skin fixed face up to the receiving cell, with PBS (pH 7.4) kept constantly at 37 °C as the receiving medium [77]. The microneedle arrays were applied to the isolated skin and samples were taken from the receiving cell at set intervals. For measuring in vitro drug release, microneedles are placed in PBS (pH 7.4, 37 °C), and samples are taken at set intervals to determine drug concentrations. Skin permeation of drugs can also be evaluated by in vivo animal models, often in suitable rats or mice. The hair of the anaesthetized animal is removed and the skin is then punctured using a microneedle patch, whilst other parameters associated with drug efficacy can be assessed, such as the microneedle strength, permeation efficiency and irritation [78].

It has been noted that skin structure and immune responses in animal models differ significantly from those in humans. In addition, the biochemical properties of ex vivo human skin are different compared to in vivo human skin [79]. Therefore, human trials need to be included in the study when conducting pharmacodynamic studies [80].

### 4.6. Biosafety and Stability

Biosafety and stability are two important issues that limit the widespread use of HFMs. One of the safety aspects of HFM systems for clinical use is biocompatibility. To ensure that HFM products are acceptable for human exposure, several tests are required to assess their biocompatibility, based on exposure times of less than 24 h, 24 to 30 h, and more than 30 h [81]. Cytotoxicity, sensitization, irritation, intracutaneous reactivity tests, genotoxicity and subacute/subchronic systematic toxicity tests are recommended for the different periods of HFM use [81].

The hemolytic assay is one of the early ways to assess toxicity [82]. Elim et al. used red blood cells from rats to evaluate the hemolytic of HFMs, and no hemolytic was observed, indicating that the materials used were haemocompatible [40]. Vicente-Perez et al. investigated the effect of repeated application HFMs arrays prepared by Gantrez^®^ S-97 BF and polyethylene glycol in the mouse skin, which displayed mild erythema, but did not stimulate the humoral immune system or cause infection or trigger an inflammatory response cascade [83]. Al-Kasasbeh et al. demonstrated for the first time in human volunteers that repeat HFM application and wear did not induce prolonged skin reactions or prolonged disruption of skin barrier function. Importantly, concentrations of specific systemic biomarkers of inflammation (C-reactive protein (CRP); tumor necrosis factor-α (TNF-α)), infection (interleukin-1β (IL-1β), allergy (immunoglobulin E (IgE)) and immunity (immunoglobulin G (IgG)) were all recorded over the course of this fixed study period. No biomarker concentrations above the normal, documented adult ranges were recorded over the course of the study, indicating that no systemic reactions were initiated in volunteers [84].

The stability of HFMs can be evaluated to ensure that active ingredients are protected during storage. This is usually done by storing HFMs and their cargo at various temperatures, including −25 ℃, 4 ℃, 20 ℃, 40 ℃ and 60 ℃, followed by analytical assessments. Generally, the protein cargo of HFMs has better storage stability and a longer shelf-life due to the rigid glassy microneedle matrices restraining molecular mobility and limiting access to atmospheric oxygen. Water should be particularly focused when non-vacuum storage conditions are present, as they can not only destroy the stability of cargo but also the mechanical properties of the HFMs themselves [85].

## 5. Application of HFMs in Disease Treatment

HFMs have been widely used for the treatment of various diseases, such as cardiovascular diseases, metabolism-related diseases and cancer, due to their outstanding advantages mentioned above.

### 5.1. Anticancer

Using HFMs for transdermal anticancer drug delivery can overcome the disadvantages of low bioavailability and side effects of oral administration and can also be used for the local administration of drugs for the treatment of superficial tumors such as melanoma, improving bioavailability while avoiding systemic exposure of the drug. Taking advantage of the abundant immune cells including antigen-presenting cells and Langerhans cells in the epidermis and dermis, the activation of the skin’s immune microenvironment can act synergistically with the drugs delivered by HFMs.

**Figure 4:**
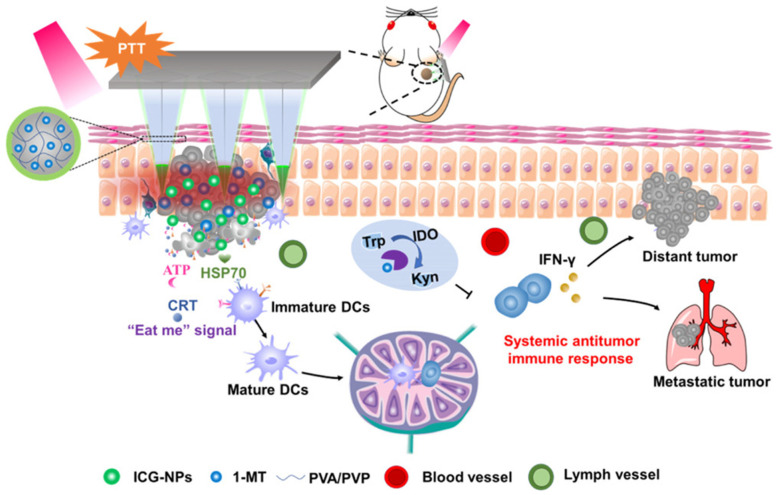
Schematic illustration of the mechanism of antitumor immunity. The figure was cited from ref. [61] with permission from the American Chemical Society.

Chen et al. prepared HFMs with cross-linking polyvinylpyrrolidone (PVP) and PVA as a matrix, loaded with 1-methyltryptophan and indocyanine green-encapsulated nanoparticles for the treatment of melanoma [86]. This system successfully induced immunogenic cell death, enhanced immune response and provided a promising melanoma treatment (Figure 4).

**Figure 5:**
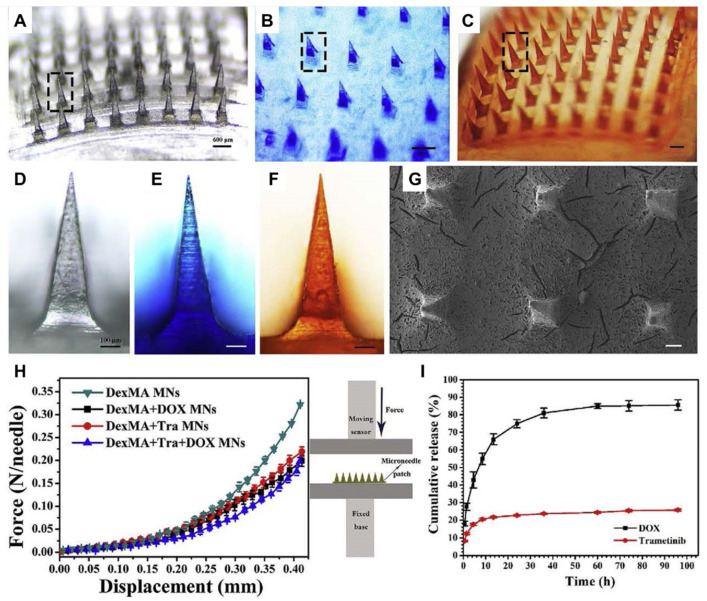
Characterization of DexMA hydrogel microneedles (MNs). (**A**–**C**) Images of blank MNs, methylene blue-loaded MNs, and DOX-loaded MNs, respectively. (**D**–**F**) Images of a single microneedle corresponding to ((**A**–**C**), marked with a dotted box), respectively. (**G**) SEM image of MNs. (**H**) MNs mechanical property. (**I**) In vitro drug release from MNs. The figure was adopted from ref. [87] with permission from Elsevier, Limited.

Huang et al. prepared HFMs loaded with doxorubicin (DOX) and trametinib (Tra) using photo-cross-linked dextrose methacrylate (DexMA) as the microneedle matrix and successfully achieved the slow release of the drugs and exploited the synergistic effect of DOX and Tra (Figure 5) [87].

### 5.2. Treating Diabetes

HFMs are a promising drug delivery system for the treatment of diabetes because they are minimally invasive, painless, have no microneedle matrix residue and can be repeatedly administered multiple times.

Chen et al. used silk protein and phenylboronic acid/acrylamide as a microneedle matrix loaded with insulin to prepare glucose-responsive smart HFMs [88]. After the microneedles penetrated the skin, insulin was released autonomously to control the blood glucose concentration when the glucose concentration in the skin tissue increased. The HFMs also retain their original needle shape after a week in water, offering the potential for safe, residue-free and sustained drug release.

**Figure 6:**
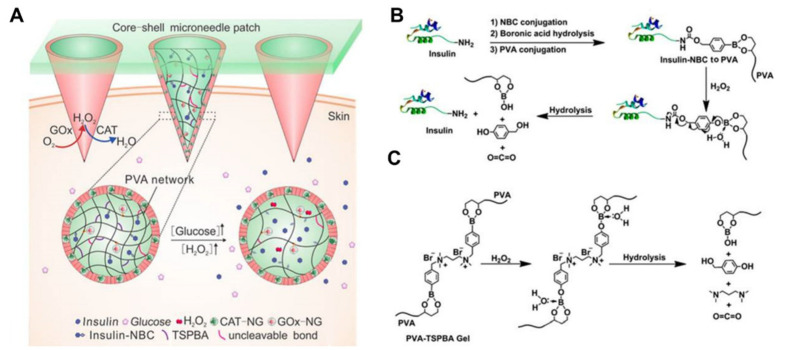
Schematic representation of the glucose-responsive insulin delivery system using H_2_O_2_-responsive PVA-TSPBA gel. (**A**) Insulin is triggered to release by a hyperglycemic state from the core matrix of the PVA-TSPBA MN patch, and the local inflammation can be greatly reduced by the catalase-embedded PVA-TSPBA shell. (**B**) Modification of insulin with NBC and H_2_O_2_-responsive release. (**C**) H_2_O_2_ responsiveness mechanism. The figure was adopted from ref. [89] with permission from the American Chemical Society.

Wang et al. fabricated HFMs by using PVA as a microneedle matrix, loaded with glucose oxidase (core) and catalase (shell) and loaded 4-nitrophenyl 4-(4,4,5,5-tetramethyl-1,3,2-dioxol-2yl) benzyl carbonate (insulin NBC) modified insulin into PVA, and PVA was further gelated by an H_2_O_2_-labile linker: N^1^-(4-boronobenzyl)-N^3^-(4-boronophenyl)-N^1^,N^1^,N^3^,N^3^-tetramethylpropane-1,3-diaminium (TSPBA) [89]. When microneedles were exposed to high glucose concentrations, local high levels of H_2_O_2_ were produced and insulin NBC was oxidized and hydrolyzed, leading to the rapid release of free insulin and control of blood glucose concentrations (Figure 6).

### 5.3. Treating Rheumatoid Arthritis

Rheumatoid arthritis is a systemic disease involving multiple joints. Early drug treatment is mostly oral administration, but long-term oral anti-rheumatoid arthritis medication often brings about serious side effects, while local injection drug treatment methods not only require specialist handling but may also pose the risk of joint damage and infection. HFMs are suitable for the delivery of drugs for this disease because of their painless and minimally invasive delivery of various active molecules.

Cao et al. designed modified hyaluronic-acid-fabricated HFMs, loaded with reverse deoxythymidine and cholesterol-modified deoxythymidine, which had a protective effect against cartilage/bone erosion in mice joints [90]. Compared to dissolving microneedles, HFMs not only increased the loading of nucleic acid aptamers into the cavity of the microneedle mold but also allowed the loading of HFMs to be controlled by adjusting the concentration of the aptamer solution, avoiding waste and loss of aptamers during preparation.

## 6. Summary and Outlook

As a new type of TDD method, HFMs have been increasingly researched for TDD, mainly in the treatment of cardiovascular diseases, tumors and diabetes mellitus, with the greatest advantage being the controlled release of the drug and the targeted drug delivery in the original lesion. In addition, the HFMs are prepared with certain functional modifications, such as some photothermal materials (e.g., gold nanorods, Prussian blue, and indocyanine green) and photosensitizers (e.g., protoporphyrin, zinc titanocyanine, and titanium dioxide), which induce the HFMs to produce exogenous stimuli (changes in temperature, magnetic fields, and light) or endogenous stimuli (changes in pH, enzymes, and redox gradients), while combining the delivered drug molecules, antibodies, nucleic acids, etc., to achieve targeted treatment of diseases. However, HFM development still faces potential biosafety issues during prolonged use, batch industrial production and sterilization challenges, which poses a huge challenge for their clinical application and is a hot topic for future research.

## Figures and Tables

**Table 1:** Microneedle products approved by the FDA.

Products Name	Applicant	De Novo or 510 (k) Number	Date Approved	Design of the Product	Use
DP4 Micro needling device	Equipmed USA LLC, Newport Beach, CA, USA	K221070	20 December 2022	Powered MN device with 16 stainless steel microneedle plate adapts to the skin’s surface and has a maximal needle length of 3 mm.	Aesthetic use
SkinPen Precision system	Crown Aesthetics, Dallas, TX, USA	K220506	7 March 2022	The microneedling pen handpiece comes with a sterile needle cartridge and includes 14 total solids medical-grade stainless steel, maximum needle length of 1.5 mm.	Aesthetic use
		K202243	2 April 2021		
INTRAcel RF Microneedle System	Jeisys Medical Inc., Seoul, Republic of Korea	K183284	15 January 2020	Fractional radio frequency combined with insulated microneedling	General and plastic surgery
Exceed Microneedling device	MT. DERM GmbH, Berlin, Germany	K182407	19 July 2019	Powered microneedling device with 6 stainless steel microneedle plate adapts to the skin’s surface and has a maximal needle length of 1.5 mm.	Aesthetic use
		K180778	7 September 2018		
SkinPen precision system	Bellus Medical, LLC, Lindon, UT, USA	DEN160029	1 March 2018	Microneedling pen handpiece with a sterile needle cartridge	Aesthetic use
MICRONJET 600	NANOPASS TECHNOLOGIES LTD., Ness Ziona, Israel	K092746	3 February 2010	Hollow microneedles that consist of a needle holder, a needle tube, a needle tube liner and a protective sleeve	Intradermal injection

**Table 2:** Microneedle products approved by the FDA for clinical trials.

Number of Clinical Trials	Study Title	Conditions	Interventions	Study Phase	Status
00837512	Insulin Delivery Using Microneedles in Type 1 Diabetes	Type 1 Diabetes Mellitus	Device: Microneedle; Device: Subcutaneous insulin catheter	Phase 2 Phase 3	Completed
03855397	Pain and Safety of Microneedles in Oral Cavity	Oral Cavity Disease	Other: Microneedle Other: Hypodermic needle Other: Flat patch	Not Applicable	Completed
04583852	Evaluate the Efficacy and Safety of Brightening Micro-needle Patch on Facial Solar Lentigines	Solar Lentigines	Other: AIVÍA, Ultra-Brightening Spot Microneedle Patch Other: Placebo Micro-needle Patch	Not Applicable	Completed
05108714	Intradermal Lidocaine Via MicronJet600 Microneedle Device	Local Anaesthesia	Device: Intravenous cannulation after intradermal injection of lidocaine via MicronJet600 microneedle device (1) Device: Intravenous cannulation after intradermal injection of saline via MicronJet600 microneedle device Device: Intravenous cannulation after intradermal injection of lidocaine via MicronJet600 microneedle device (2) Procedure: Intravenous cannulation after without prior interventions	Not Applicable	Completed
05267938	Microneedle Pretreatment as a Strategy to Improve the Effectiveness of Topical Anesthetics Formulations	Oral Cavity Disease	Drug: Topical Anesthetic Drug: Local anesthetic	Phase 1	Completed
04989361	Soluble Hyaluronic Acid Microneedle VS. Non-ablative Fractional Laser on Infraorbital Wrinkles	Wrinkle	Procedure: Soluble Hyaluronic Acid Microneedle Procedure: Nonfractional laser	Not Applicable	Recruiting
05377905	Microneedle Array Plus Doxorubicin in Cutaneous Squamous Cell Cancer (cSCC)	Cutaneous Squamous Cell Carcinoma Skin Cancers-Squamous Cell Carcinoma	Drug: Microneedle Array Doxorubicin (MNA-D)	Phase 1 Phase 2	Not yet recruiting
02966067	A Split Mouth Trial to Compare Microneedles vs. Standard Needles in Dental Anaesthetic Delivery	Dental Pain Anesthesia, Loca	Device: Microneedle Device (Experimental) Device: 30-gauge Short Hypodermic Needle	Not Applicable	Completed
03054480	Fractional Micro-Needle Radiofrequency and I Botulinum Toxin A for Primary Axillary Hyperhidrosis	Primary Axillary Hyperhidrosis	Device: Fractional Micro-Needle Radiofrequency Drug: Botulinum toxin type A	Not Applicable	Completed
03207763	Microneedle Patch Study in Healthy Infants/Young Children	Vaccination Skin Absorption	Device: Microneedle Formulation 1 Device: Microneedle Formulation 2	Not Applicable	Completed
02682056	Glucose Measurement Using Microneedle Patches	Diabetes	Device: Microneedle patch Device: Intravenous (IV) catheter Device: Lancet	Not Applicable	Completed
04732195	Pilocarpine Microneedles for Sweat Induction (PMN-SI)	Cystic Fibrosis	Device: Pilocarpine microneedle patch Device: Pilocarpine Iontophoresis	Not Applicable	Completed
05694858	Transdermal Microneedle Lignocaine Delivery Versus EMLA Patch for Topical Analgesia Before Venepuncture Procedure To Adults in Clinical Setting	Glaucoma Cataract	Combination Product: Lignocaine loaded maltose microneedle array patch Drug: EMLA 5% patch	Phase 1 Phase 2	Not yet recruiting
02995057	Safety Demonstration of Microneedle Insertion	Allergic Reaction to Nickel	Device: Gold- or silver-coated, or uncoated nickel microneedles	Not Applicable	Completed
04552015	Microneedles for Diagnosis of LTBI	Latent Tuberculosis	Diagnostic Test: TST vs. PPD microneedle test	Not Applicable	Terminated
03203174	The Use of Microneedles With Topical Botulinum Toxin for Treatment of Palmar Hyperhidrosis	Hyperhidrosis	Device: Microneedle Device: Sham Microneedle Drug: Botulinum Toxin Type A Other: Saline	Phase 1	Completed
03332628	Racial/Ethnic Differences in Microneedle Response	Healthy	Device: Microneedle patch	Not Applicable	Completed
05078463	Efficacy of Transdermal Microneedle Patch for Topical Anesthesia Enhancement in Paediatric Thalassemia Patients	Thalassemia in Children	Device: Microneedle Drug: 1 Finger Tip Unit (FTU) EMLA Cream (30-min application time) Drug: 1 Finger Tip Unit (FTU) EMLA (15-min application time) Drug: 0.5 Finger Tip Unit (FTU) EMLA (30-min application time) Device: Sham Patch	Phase 2	Completed
00539084	A Study to Assess the Safety and Efficacy of a Microneedle Device for Local Anesthesia	Local Anesthesia Intradermal Injections	Device: MicronJet	Not Applicable	Completed
02596750	The Effect of Microneedle Pretreatment on Topical Anesthesia	Pain	Device: Microneedle Roller Device: Sham microneedle Roller	Not Applicable	Completed
01812837	The Use of Microneedles in Photodynamic Therapy	Actinic Keratosis	Device: Microneedle Drug: Aminolevulinic Acid Radiation: Blue light	Not Applicable	Completed
05710068	Effects of RF Microneedle on Photoaging Skin	Pigmentation Pigmentation Disorder	Device: RF Microneedle Drug: Combination cream	Not Applicable	Completed
03629041	A Study of the Use of Microneedle Patches to Deliver Topical Lidocaine in the Oral Cavity	Topical Anaesthesia	Device: Microneedle Patch Device: Patch with no microneedles	Phase 1	Completed
03795402	Analysis of Non-Invasively Collected Microneedle Device Samples From Mild Plaque Psoriasis for Use in Transcriptomics Profiling	Psoriasis Vulgaris	Device: Microneedle Device	Not Applicable	Completed
02594644	The Use of Microneedles to Expedite Treatment Time in Photodynamic Therapy	Keratosis, Actinic	Device: Microneedle Roller Drug: Aminolevulinic Acid Radiation: Blue Light	Not Applicable	Completed
03415373	Clinical Evaluation of Healthy Subjects Receiving Intradermal Saline Using the Microneedle Adapter (Model UAR-2S)	Intradermal Injection	Device: Microneedle Adapter (Model UAR-2S) Device: Hypodermic needle + syringe	Not Applicable	Completed
03607903	Adalimumab Microneedles in Healthy Volunteers	Pain Injection Site	Biological: Adalimumab ID Biological: Adalimumab SC Other: Saline ID Other: Saline SC	Phase 1 Phase 2	Completed
04394689	Measles and Rubella Vaccine Microneedle Patch Phase 1–2 Age De-escalation Trial	Measles Rubella Vaccination Healthy	Biological: Measles Rubella Vaccine (MRV-SC) Biological: MRVMNP Other: PLA-MNP Other: PLA-SC	Phase 1 Phase 2	Completed
03739398	A Study on the Effectiveness and Safety Evaluation of Combination Therapy With 1927 nm Thulium Laser and Fractional Microneedle Radiofrequency Equipment for Improvement of Skin Aging	Wrinkle	Device: LUTRONIC GENUS laser (Fractional Microneedle Radiofrequency (FMR)) Device: LASEMED laser (The Thulium laser with 1927 nm wavelength)	Not Applicable	Completed
02438423	Inactivated Influenza Vaccine Delivered by Microneedle Patch or by Hypodermic Needle	Influenza	Biological: Inactivated influenza vaccine Other: Placebo	Phase 1	Completed
04928222	Placebo Microneedles in Healthy Volunteers (Part I) and Efficacy/Safety of Doxorubicin Microneedles in Basal Cell Cancer Subjects (Part II)	Basal Cell Carcinoma	Combination Product: Doxorubicin containing MNA Drug: Placebo containing MNA	Phase 1 Phase 2	Active, not recruiting
02632110	Microneedle Lesion Preparation Prior to Aminolevulinic Acid Photodynamic Therapy (ALAPDT) for AK on Face	Actinic Keratosis	Drug: ALA Drug: Topical Solution Vehicle Device: IBL 10 mW Procedure: Microneedle lesion preparation Device: IBL 20 mW	Phase 2	Completed
02745392	Safety and Efficacy of ZPZolmitriptan Intracutaneous Microneedle Systems for the Acute Treatment of Migraine	Acute Migraine	Drug: ZPZolmitriptan Drug: Placebo	Phase 2 Phase 3	Completed
01789320	Safety Study of Suprachoroidal Triamcinolone Acetonide Via Microneedle to Treat Uveitis	Uveitis Intermediate Uveitis Posterior Uveitis Panuveitis Noninfectious Uveitis	Drug: triamcinolone acetonide (Triesence^®^)	Phase 1 Phase 2	Completed

**Table 3:** Characteristics of different HFM formulations.

Compounds	Polymer	Characteristics	Ref.
Fluorescein, FITC-Dextran, Doxorubicin	Methacrylated hyaluronic acid	Methacrylated hyaluronic acid microneedles fully swelled within 1 min, with swelling ratio of ~2.74; >80% of fluorescein, FITC-Dextran, and >50% of doxorubicin were released from the microneedle patches within 30 min.	[37]
HRP	Silk	The beta sheet content in the microneedle devices was increased from 14% to 15% and 21% as the water vapor annealing time increased from 0 h to 2 h and 8 h, and the HRP release reduced to 37% and 18%.	[38]
FITC-dextran	Silk fibroin, urea, N-dimethylformamidee, glycine and 2-ethoxyethanol	The swelling-modified silk fibroin microneedles with different microscopic pore size attains 250–650% swelling ratio after PBS immersion; the swelling-modified silk fibroin microneedles display significantly enhanced transdermal drug release kinetics compared with the controlled silk fibroin films, with 2–10-times larger accumulative release ratio than the corresponding control groups during the entire release process in vitro.	[2]
Light-responsive ibuprofen conjugates	Crosslinked 2-hydroxyethyl methacrylate	The crosslinked 2-hydroxyethyl methacrylate hydrogel shows maximum swelling degrees of around 50% after 24 h; the system allows the release of ibuprofen during prolonged periods of time (up to 160 h).	[39]
Sildenafil citrate	Polyvinyl alcohol and polyvinylpyrrolidone crosslinked by tartaric acid	The hydrogel’s swelling percentage was 348.07–72,897% with different formulations.	[40]
Doxorubicin	Methacrylated hyaluronic acid	The swelling ratio increased rapidly and reached a maximum of 337% at 10 min; the release profile of doxorubicin/SMNs dramatically turned into a slow rate after 90 min and less than 90% doxorubicin was released at the end of the detection point (12 h). The maximum doxorubicin concentration that appeared at 1 h was 0.58 ± 0.35 μg/mL for the doxorubicin/DMNs group, and was 1.28 ± 0.32 μg/mL for the doxorubicin/SMNs group at 2 h, respectively. The crosslinking network of SMNs significantly retarded the diffusion of small molecule drugs within the needle matrix, and extended the drug release duration, increasing the drug transdermal efficacy.	[41]
Nicotinamide mononucleotide	Polyvinyl alcohol, carboxymethyl cellulose, DMSO	Formulations of microneedles containing 2.9% carboxymethyl cellulose have a higher swelling ratio (186%) in comparison with the 0% carboxymethyl cellulose composite (48%) and a higher nicotinamide mononucleotide release of 91.94 *±* 4.03% at 18 h compared with the carboxymethyl cellulose-free polyvinyl alcohol matrix of 50.48 *±* 3.73% at 18 h.	[42]

## Data Availability

Not applicable.
